# Endogenous *gpdh-1* transcriptional reporters as new tools for the study of the osmotic stress response

**DOI:** 10.17912/micropub.biology.000818

**Published:** 2023-03-22

**Authors:** María Victoria Veroli, Todd Lamitina

**Affiliations:** 1 Department of Pediatrics, University of Pittsburgh, Pittsburgh, Pennsylvania, United States

## Abstract

*In vivo* monitoring of *gpdh-1* gene expression using standard transcriptional reporters is a powerful and commonly used tool for genetic dissection of the osmotic stress response in *C. elegans* . Like all transgene reporters, these *gpdh-1* reporters have important limitations that restrict their utility. To overcome these limitations, we created three different *gpdh-1* reporters using CRISPR/Cas9 methods to insert several variants of GFP into the endogenous *gpdh-1* locus. These new strains provide a more powerful and accurate tool for the analysis of *gpdh-1* regulatory pathways.

**Figure 1. Endogenous GFP reporters provide precise and quantitative information on gpdh-1 expression and localization:**
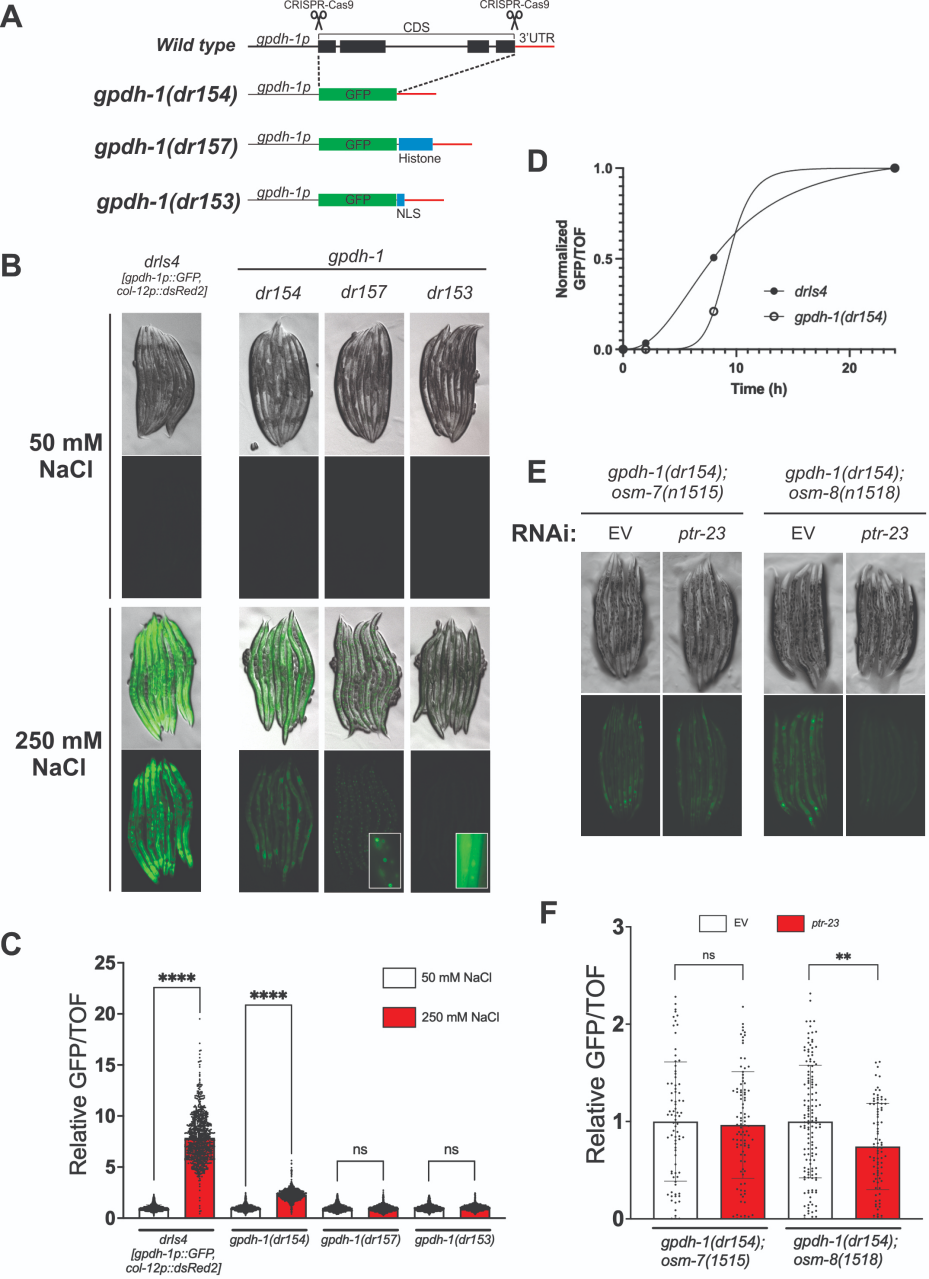
A) Design strategy for the endogenous *gpdh-1* reporters. These consist on the substitution of the whole *gpdh-1* coding sequence (CDS) to generate *gpdh-1 (dr153)* (GFP::NLS), *gpdh-1 (dr154)* (GFP), or *gpdh-1 (dr157)* (GFP- *his-58* ). B) Images of day1 adult animals expressing the indicated *gpdh-1* reporter under normal (50 mM NaCl) and hypertonic (250 mM NaCl) conditions. The *drIs4* reporter is shown as reference. All images were taken using the same exposure settings. Insets in *dr157* and *dr153* are longer exposures and show that these reporters express at low levels and give rise to nuclear enriched GFP signal in the hypodermis and intestine under hypertonic conditions. C) Relative fold induction of GFP intensity by hypertonic conditions (250 mM NaCl) for the reporters shown in B. Data is represented as mean ± S.D. ****p<0.0001, ns: nonsignificant. (Mann-Whitney test). Data were normalized to the mean expression levels at 50 mM NaCl for each genotype. D) Kinetics of *gpdh-1 (dr154)* and *drIs4* activation measured as the change in normalized GFP/TOF within 0, 2, 8 and 24h. Data was fit to a four parameter logistic (4PL) curve. N= 800-3000 for each data point. E) Images of *osm-7 ( n1515 ); gpdh-1 (dr154)* or *osm-8 ( n1518 ); gpdh-1 (dr154)* day 1 adults following 2 generation exposure to *ptr-23 (RNAi)* . All images were taken using the same exposure time. F) COPAS quantification of the relative fold changes of GFP intensity of *osm-7 ( n1515 );* *gpdh-1 (dr154)* and *osm-8 ( n1518 ); gpdh-1 (dr154)* exposed to either empty vector (EV) or *ptr-23 (RNAi).* Data for each genotype is normalized to the EV mean and represented as mean ± S.D. **p<0.005, ns: nonsignificant. (Mann-Whitney test). N=81-139 for each condition.

## Description

Increased expression of the *gpdh-1* gene in *C. elegans* intestine and hypodermis is a robust and specific reporter of hypertonic stress response activation. Transgenic *gpdh-1 p::gfp* reporters are commonly used for large-scale genetic screens to identify positive and negative regulators of the hypertonic stress response, as well as for more focused mechanistic studies (Lamitina *et al.* 2006; Rohlfing *et al.* 2010; Rohlfing *et al.* 2011; Lee and Strange 2012; Dodd *et al.* 2018; Urso *et al.* 2020; Chandler and Choe 2022; Wimberly and Choe 2022). However, there are limitations associated with the existing *gpdh-1* reporters. These include the use of incomplete *gpdh-1* promoter sequences, absence of other native regulatory sequences (i.e. 3’ UTR), overexpression, and non-native chromosomal context, which might preclude the identification of bonafide *gpdh-1* regulators that function through such regulatory regions. Additionally, the absence of such regulatory sequences might provide an incomplete description of the native expression pattern of *gpdh-1* . One example of this is the lack of expression of overexpressed transgenes in the germline (Kelly *et al.* 1997). To address these limitations, we generated a new *gpdh-1* transcriptional reporter using CRISPR/Cas9 to replace the endogenous *gpdh-1* coding sequence with that of GFP.

We were concerned that a single copy *gpdh-1 p::GFP* reporter might not be detectable under stereo fluorescence microscopy, which would severely limit its utility. To potentially increase the GFP signal, we created several different GFP repair templates, including versions in which GFP contained either a nuclear localization signal (NLS) or the *his-58* DNA binding histone sequence to concentrate the GFP signal in the nucleus ( **Figure 1A** ). While all of these approaches were successful ( **Figure 1B,C** ), we found that the simple GFP knock-in gave the most robust signal. As expected, the induction was significantly less than that observed using the overexpression reporter *drIs4* , although the kinetics and sensitivity of both reporters were similar ( **Figure 1D** ). Despite the reduced expression level in response to hypertonicity, the native *gpdh-1 p::GFP* reporter was easily observed by eye under stereo fluorescence microscopy and generated a robust signal on the COPAS Biosort. However, the nuclear-localized GFP lines were not easily observed on the stereomicroscope and were not detectable on the COPAS Biosort ( **Figure 1B,C** ). The expression pattern of all of the lines showed robust GFP induction in only two tissues – the intestine and the hypodermis, indicating that the overexpression reporters do provide an accurate spatial representation of endogenous *gpdh-1* expression. The new reporter also recapitulated previously described mutant phenotypes. For example, in the *osm-8 ( n1518 )* mutant (Rohlfing *et al.* 2010), endogenous *gpdh-1 (dr154)* reporter animals exhibited very high GFP expression in the absence of hypertonicity ( **Figure 1E,F** ). After growing *osm-8 ( n1518 )* on *ptr-23 (RNAi)* , GFP was reduced, as previously shown (Rohlfing *et al.* 2011). While we found that *osm-7 ( n1515 )* animals also exhibited elevated GFP expression from *gpdh-1 (dr154)* , we found that *ptr-23 (RNAi)* did not reduce GFP expression in *gpdh-1 (dr154); osm-7 ( n1515 )* animals ( **Figure 1E,F** ), suggesting *osm-7* and *osm-8* act in distinct genetic pathways to regulate *gpdh-1* expression.

The properties of this new *gpdh-1 p::GFP* reporter make it a better tool for uncovering genetic regulators of the hypertonic stress response pathway as compared to previously used overexpression reporters. Nevertheless, this new tool does still have some limitations. Since the GFP insert replaces the *gpdh-1* coding sequence, the *gpdh-1 (dr154)* allele is a *gpdh-1* null. Even though *gpdh-1* nulls are viable, fertile, and only have a minor hypertonic stress adaptation phenotype (Urso *et al.* 2020), the lack of *gpdh-1* gene expression in these reporter animals could potentially alter physiological feedback loops regulating osmosensitive gene expression. However, our findings that *osm-7* and *osm-8* mutants, as well as *ptr-23 (RNAi)* , alter GFP expression levels from *gpdh-1 (dr154)* similarly to the *drIs4* overexpression reporter, which contains a wild type *gpdh-1* gene, suggests this is not a major problem. An obvious solution to further improve the accuracy of *gpdh-1* reporters is to tag the endogenous *gpdh-1* coding sequence. However, our first attempt to generate a C-terminally tagged *gpdh-1* allele did not generate a visible GFP signal, even though we were able to detect GPDH-1 ::GFP protein biochemically (Urso *et al.* 2020). This lack of signal may be caused by the localization of the GPDH-1 ::GFP protein into a subcellular compartment that quenches GFP fluorescence, such as acidic lysosomes. Tagging GPDH-1 with more pH-stable fluorophores, such as mCherry or other RFP variants, might overcome this challenge (Roberts *et al.* 2016).

## Methods

**CRISPR/Cas9 genomic editing.** CRISPR alleles were generated using methods adapted from (Dokshin *et al.* 2018). We generated CRISPR microinjection mixes (20 µl total volume) containing 0.25 µg/µl purified Cas9 (IDT), 20 ng/µl tracrRNA (IDT), and 20 ng/µl guide RNA (IDT). This mix was incubated at 37ºC for 10 minutes. Separately, we took 200 ng of repair template generated via High-Fidelity PCR (Q5 polymerase NEB; primers contain SP9 modification of 5’ end) and ran it through a melt-reanneal protocol (95ºC - 2:00 min; 85ºC - 10 sec, 75ºC - 10 sec, 65ºC - 10 sec, 55ºC - 1:00 min, 45º - 30 sec, 35º - 10 sec, 25º 10 sec, 4º hold; 1ºC/second ramp). To the Cas9 mix, we added 200 ng of melted/reannealed DNA (20 ng/µl final) and *rol-6* plasmid DNA (40 ng/µl). Day 1 adult N2 animals were injected and 24 F1s from each of 2-3 P0 injected animals with >15-20 rollers were singled. The progeny of each F1 were screened for the presence of the GFP insert using PCR and homozygous insert bearing animals were identified through additional PCR screening. gRNA and primers are found in Reagents section.

**Imaging and COPAS analysis** . Day one adults from a synchronized hypochlorite preparation were seeded on 50 or 250 mM NaCl NGM plates. After 24 hours, images were captured using M205FA fluorescence dissecting scope and a DFC345FX digital camera using identical exposure times for the different strains in Leica Advanced Fluorescence Software, v2.1.0. For *gpdh-1 ::GFP* *dr153* and *dr157* CRISPR alleles, GFP images were also captured with a 64X lens and GFP filter (Leica L5 ET set) on a DMI4000B with a DFC 340FX camera. GFP fluorescence intensity and time of flight (TOF) of each animal were acquired with the COPAS Biosort. Events in which the TOF was < 400 (worms younger than adults) and < 20 (dead worms or other objects) were excluded from the analysis. The GFP fluorescence intensity of each animal was normalized to its TOF and divided by the average GFP/TOF of that strain exposed to 50 mM NaCl to determine the fold induction of GFP for each animal.

## Reagents

*C. elegans* Strains:

**Table:** 

**Strain**	**Genotype**
N2	*wild type*
OG119	*drIs4* [ *gpdh-1 p* :: *GFP; col- 12p* :: *dsRed2* ]
OG1263	*gpdh-1 (dr153) [ gpdh-1 ::GFP-NLS] *
OG1264	*gpdh-1 (dr154) [ gpdh-1 ::GFP] *
OG1269	*gpdh-1 (dr157) [ gpdh-1 ::GFP-His58] *

DNA oligos:

**Table:** 

**Primer name**	**Sequence**	**Description**
OG2039	CAGCTCGTCAATAAAACACATAAAACTATCACTTA TTTGTATAGTTCATCCATGC	*gpdh-1 (dr153 and dr154)* repair template amplification (antisense)
OG2040	GAATCAGATTTTTACCCATATCACAACAACTTATT ATGACTGCTCCAAAGAAGAAG	*gpdh-1 (dr153)* repair template amplification (sense)
OG1544	ACTATCACTTA ATCATACTC TGG	*gpdh-1* CRISPR alleles C-term gRNA (antisense)
OG2037	GAATAGTCAAAGTTCTGGCA CGG	*gpdh-1* CRISPR alleles N-term gRNA (antisense)
OG1551	AGTCGATCAATCAGGGACTCG	*gpdh-1* alleles genotyping (antisense)
OG1556	GCATTCATCAAGAGTGATAAGGTAGG	*gpdh-1 (dr153 and dr154)* genotyping (sense)
OG2041	CGATGGCCCTGTCCTTTTACCAG	*gpdh-1 (dr153 and dr154)* genotyping (sense)
OG2038	GAATCAGATTTTTACCCATATCACAACAACTTATT ATGAGTAAAGGAGAAGAACT	*gpdh-1 (dr157 and dr154)* repair template amplification (sense)
OG2117	CCAGCTCGTCAATAAAACACATAAAACTATCACTTA CTTGCTGGAAGTGTACTTG	*gpdh-1 (dr157)* repair template amplification (antisense)
OG1944	GGGTAAGTTTTCCGTATGTTGCA	*gpdh-1 (dr157)* genotyping (antisense)
OG2042	TCTAGATTCCGCGAATCTCTG	*gpdh-1 (dr157)* genotyping (sense)
OG2118	GTCTCCTCCAAGGCCATG	*gpdh-1 (dr157)* genotyping (sense)
